# Scaling COPC in South Africa: Insights and Priorities from the 2024 National Workshop

**DOI:** 10.4102/phcfm.v17i1.5022

**Published:** 2025-12-03

**Authors:** Karessa Govender, Helen Schneider, Lucy Gilson, Robert Mash, Eleanor Whyle, Oupa Motshweneng, Nonhlanhla Nkosi, Charlyn Goliath, Hassan Mahomed, Saadiq Kariem

**Affiliations:** 1 School of Public Health, Faculty of Community and Health Sciences, University of the Western Cape, Cape Town, South Africa; 2 School of Public Health, Faculty of Health Sciences, University of Cape Town, Cape Town, South Africa; 3 Department of Family and Emergency Medicine, Faculty of Health Sciences, Stellenbosch University, Cape Town, South Africa; 4 Western Cape Department of Health and Wellness, Cape Town, South Africa; 5 Valkenberg Hospital, Cape Town, South Africa; 6 Division of Health Systems and Public Health, Faculty of Health Sciences, Stellenbosch University, Cape Town, South Africa

## Introduction

There has been increasing interest in Community-Oriented Primary Care (COPC) in South Africa in recent years. Community-Oriented Primary Care was first introduced in KwaZulu-Natal (KZN) in the 1940s.^1^ It has re-emerged and has been implemented in varied ways and settings across the country, including in KZN, Gauteng and Western Cape. Community-Oriented Primary Care is of relevance to ongoing health reforms that seek to reorganise the South African health system and provide health care services in an equitable manner.

Community-Oriented Primary Care is defined as:

[*A*] continuous process by which primary health care is provided to a defined community on the basis of its assessed health needs, by the planned integration of primary care practice and public health.^1^

On 14 August 2024, the Western Cape Department of Health and Wellness (WCDHW), in partnership with local universities and the South African Learning Alliance for the District Health System (SALAD), held a 1-day COPC workshop as part of its Healthcare 2030 Strategy review. The event brought together 53 participants from 6 provincial health departments, 5 universities, the National Department of Health and World Health Organization’s (WHO) Alliance for Health Policy and Systems Research (AHPSR).

The workshop aimed to build consensus on COPC, clarify its relevance in the context of current health system realities across provinces, share implementation experiences and identify priorities for scaling COPC nationally addressing key gaps such as the lack of a unified national understanding of COPC models.

After an introductory input on the origins and core features of COPC, the workshop participants heard from COPC implementors across the country on their models and experiences, shared experiences with different dimensions of implementation in world café sessions and conducted group priority setting on how to advance COPC in South Africa.

The themes discussed in the world café sessions were:

- Organising the continuum: integrated care pathways.
- Training and ongoing development.
- (Re)orienting primary health care (PHC) facilities and teams.
- Beyond the household: strategies for community engagement, intersectoral collaboration.
- System-wide and system-deep enablement at sub-district, district and provincial levels.
- Monitoring, evaluation, learning (MEL).

The workshop proceedings are available at https://soph.uwc.ac.za/news/copc-workshop-report/ and are summarised as follows.

## The origin and development of Community-Oriented Primary Care in South Africa

The origin of COPC in 1940 was part of a broader strategy to provide more equitable and accessible health care services, envisaged by prominent health actors at the time as similar to the National Health Service (NHS) emerging in the United Kingdom (UK). The South African NHS would be founded on a network of PHC centres modelled after the Pholela model.^1^ Dr Sydney Kark was appointed to lead the first state-sponsored, Pholela Health Care Unit in the Harry Gwala district in KZN.^1^

The Pholela Health Care Unit aimed to provide preventative and curative services in rural areas, with the goal of scaling this nationally. It used a population-based approach, integrating public health and primary care. Key aspects included health education, health promotion, community participation and empowerment, understanding the population and addressing social determinants of health. To reach more people, community members were trained as health assistants and educators, focusing on empowering families to improve their health. Health workers were required to understand the health needs of everyone in a household.

This programme formed part of the early beginnings of the PHC movement ultimately leading to the Alma Ata Declaration.^1^ The Pholela programme was shut down after 6 years in 1948 by the new apartheid political dispensation.^2^ However, COPC remained an enduring idea in South Africa and gained traction in several parts of the country following the adoption of the primary care reengineering strategy and the introduction of National Health Insurance (NHI).^3^

## Models of Community-Oriented Primary Care in South Africa

### KwaZulu-Natal’s community-based model

Hlobisile Langa (Acting Director for Primary Health Care and District Health Services in KZN) presented KZN’s community-based model (CBM).

Community-based model is based on the original Pholela Health Unit Model, which was revived in 2018. It is grounded in health prevention and promotion. Key aspects include community participation and empowerment, with a focus on the health of the household as a unit. The CBM has been adopted in all 11 districts in KZN, covering 12.8 million people.

Community health workers (CHWs) are organised into Ward-Based Primary Healthcare Outreach Teams (WBPHCOTs) and School Health Teams (SHTs) and are supported by operational managers (OMs) and PHC managers based at PHC facilities or sub-district offices.

The CBM is implemented through a three-part strategy:

- Operation Sukhuma Sakhe promotes coordinated service delivery through collaboration between government, communities, non-governmental organisations (NGOs) and other providers at the ward level.
- Primary health care services are decentralised to sub-districts, with district or regional hospitals and community health centres (CHCs) supporting local clinics – laying the groundwork for NHI’s contracting units for primary care (CUPs).
- Household champions, nominated family members, help detect illness early, refer to services and support chronically ill relatives.

### Community-Oriented Primary Care at Chiawelo Community Practice, Soweto

Doctor Shabir Moosa and Shivani Pillay presented the Chiawelo Community Practice (CCP) model.

**FIGURE 1:**
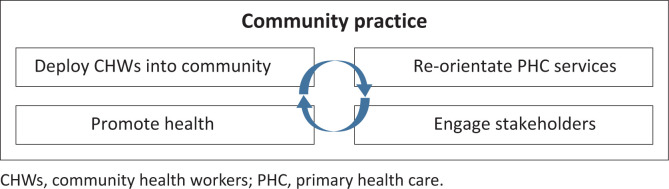
Community practice model.

The CCP model was implemented in a single ward (Ward 11) in Soweto in 2014, following the earlier release of the PHC reengineering framework in 2011 and with the goal to pilot general physician (GP)-led primary health care as a model for NHI. The CCP adopts a team approach, led by a family-physician within a broader team of CHWs and clinical associates. Using a complex adaptive systems approach, the model includes four key interventions, outlined in Figure 1. Similar to Pholela and CBM, CHWs are integral to all four interventions:

- Community health worker deployment: CHWs, led by an enrolled nurse, collect household data to build community profiles and address health issues – resolving 89% of 3139 identified problems by 2017.
- Community-Oriented Primary Care: Patients are redirected from CHCs to CCPs. Community health workers manage integrated family folders and are supported by a multidisciplinary team.
- Stakeholder engagement: Community health workers map community assets, with regular meetings held for patient groups and annual forums for broader stakeholder input.
- Targeted health promotion: Community health workers offer screening, health education during visits, run monthly health clubs and conduct campaigns on issues like drug abuse.

### University of Pretoria’s information and communication technology enabled Community-Oriented Primary Care

Prof. Jannie Hugo presented the University of Pretoria’s (UP) information and communication technology (ICT)-enabled COPC model, introduced in Tshwane in 2011 under PHC reengineering. Like the CCP model, it uses a team-based, clinician-led approach, with CHWs supported by nurses, clinical associates and family physicians.

The model is supported by AitaHealth, an ICT-enabled device and multi-functional web platform, which is interoperable with the District Health Information System (DHIS). AitaHealth identifies individual and household health care requirements and supports quality service delivery, human and material resource management, capacity building and monitoring and evaluation.^4^

The core elements of the model are (1) team, (2) training, (3) planning, (4) key activities, (5) referrals, (6) information systems and (7) governance.

Elements of the UP COPC model have been piloted in districts of Gauteng (2017–2018) and in Anglo-American mining communities in Bojanala district in the North West.

### Community-Oriented Primary Care in the Western Cape

Annie van Rensburg and Liz Pegram, representing WCDHW, shared their experiences with the implementation of COPC.

In the Western Cape, COPC was spurred by a large quadruple burden of disease and decreasing budgets.^5^ In 2014, the province developed its Health Care 2030 Strategy, which calls for a shift from a focus on curative care to disease prevention, health and well-being across the lifespan and PHC.^6^ Community-Oriented Primary Care has been adopted as a key strategy for improvement of district health services in the province and was first introduced in 2017 in eight sub-district learning sites. In 2022, the province released ‘Health is everyone’s business: A framework for action’, which prioritised COPC as key to service redesign. In addition, COPC is an integral component of preparation for the NHI CUPs.

The province adopted the core principles of COPC: (1) a defined community, (2) a multidisciplinary team approach, (3) a comprehensive approach, (4) equity, (5) person-centred care, (6) data driven and evidence-based, (7) whole of society approach and h) adopts a change management process.

The COPC implementation framework comprises ten interrelated elements: geographic delineation of PHC teams, composition of PHC teams, facility- and community-based teamwork, partnership of government and non-government organisations, scope of practice, information system, community engagement, stakeholder engagement, training and development of PHC teams, system preparation and change management.^7^

**FIGURE 2:**
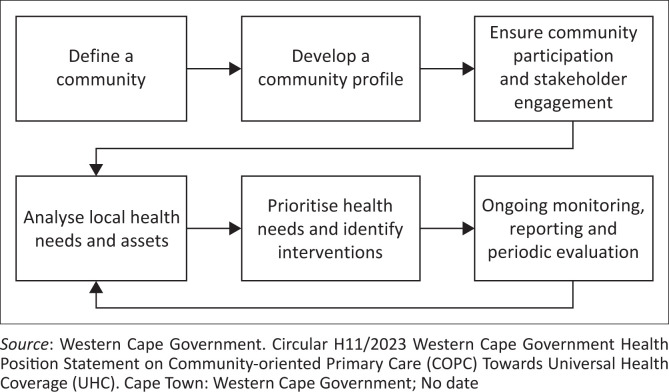
Western Cape’s Community-Oriented Primary Care process.

As illustrated in Figure 2,^8^ the Western Cape’s COPC process consists of six key steps.

Enablers of implementation include: (1) good communication, (2) access to information, (3) relationship-building, (4) resource allocation, (5) community engagement and (6) change management.

### Priorities for national Community-Oriented Primary Care development

After discussing experiences, participants brainstormed individually, grouped priorities and then voted in plenary to define national priorities.

Attendees agreed that COPC is key to NHI and Universal Health Coverage and emphasised the need for strong political will, a clear national policy framework and well-resourced, multi-level implementation strategies. Advancing COPC also requires:

- Strengthen integrated care models and pathways through multidisciplinary teams, including CHWs, clinical associates, family physicians and PHC staff.
- Provide training and ongoing support for these teams.
- Invest in monitoring, evaluation and learning (MEL) systems and digital innovations.
- Apply change management strategies using tools from existing COPC sites.
- Promote intersectoral governance through whole-of-government and whole-of-society approaches.

## Concluding reflections

Dr Aku Kwamie of the WHO’s AHPSR and Dr Keith Cloete, the Head of WCDHW, shared their reflections on the workshop.

Community-Oriented Primary Care addresses social complexity, and while hospital care may be clinically complex but socially simple, PHC is often clinically simple but socially complex, requiring skills beyond clinical training to navigate social contexts and power dynamics.

Key considerations for implementing COPC:

- Allow time for building trust and relationships essential for systems change.
- Strong facilitation and cultural competency are vital; social and relational skills are as important as technical training.
- Collaboration needs shared governance involving a broad ecosystem (PHC teams, communities, etc.); structured forums are valuable. Initiatives like KZN’s ‘War Rooms’ show the value of structured cross-sectoral forums.
- Shift from hierarchical leadership towards systems thinking and relational approaches to support collaboration on complex issues.
- Create opportunities for collective learning. Beyond formal MEL and research, informal reflection, such as Western Cape’s quarterly debriefs, uncovers tacit knowledge often missed in official reports.

Dr Kwamie stressed the need for practice-informed evidence and encouraged attendees to document their COPC implementation to balance researcher-dominated knowledge production and scholarship.
